# Paramedic Recognition of Sepsis in the Prehospital Setting: A Prospective Observational Study

**DOI:** 10.1155/2016/6717261

**Published:** 2016-03-09

**Authors:** Robert S. Green, Andrew H. Travers, Edward Cain, Samuel G. Campbell, Jan L. Jensen, David A. Petrie, Mete Erdogan, Gredi Patrick, Ward Patrick

**Affiliations:** ^1^Department of Critical Care, Dalhousie University, Suite 377, Bethune Building, 1276 South Park Street, Halifax, NS, Canada B3H 2Y9; ^2^Trauma Nova Scotia, Room 1-026B, Centennial Building, 1276 South Park Street, Halifax, NS, Canada B3H 2Y9; ^3^Department of Emergency Medicine, Dalhousie University, Division of EMS, 1796 Summer Street, Halifax Infirmary, Suite 355, Halifax, NS, Canada B3H 3A7; ^4^Emergency Health Service, 239 Brownlow Avenue, Suite 300, Dartmouth, NS, Canada B3B 2B2; ^5^Performance Excellence, Nova Scotia Health Authority, Halifax, NS, Canada B3H 4R2

## Abstract

*Background*. Patients with sepsis benefit from early diagnosis and treatment. Accurate paramedic recognition of sepsis is important to initiate care promptly for patients who arrive by Emergency Medical Services.* Methods*. Prospective observational study of adult patients (age ≥ 16 years) transported by paramedics to the emergency department (ED) of a Canadian tertiary hospital. Paramedic identification of sepsis was assessed using a novel prehospital sepsis screening tool developed by the study team and compared to blind, independent documentation of ED diagnoses by attending emergency physicians (EPs). Specificity, sensitivity, accuracy, positive and negative predictive value, and likelihood ratios were calculated with 95% confidence intervals.* Results*. Overall, 629 patients were included in the analysis. Sepsis was identified by paramedics in 170 (27.0%) patients and by EPs in 71 (11.3%) patients. Sensitivity of paramedic sepsis identification compared to EP diagnosis was 73.2% (95% CI 61.4–83.0), while specificity was 78.8% (95% CI 75.2–82.2). The accuracy of paramedic identification of sepsis was 78.2% (492/629, 52 true positive, 440 true negative). Positive and negative predictive values were 30.6% (95% CI 23.8–38.1) and 95.9% (95% CI 93.6–97.5), respectively.* Conclusion*. Using a novel prehospital sepsis screening tool, paramedic recognition of sepsis had greater specificity than sensitivity with reasonable accuracy.

## 1. Introduction

Despite numerous clinical trials of specific treatments, sepsis remains a leading cause of morbidity and mortality in patients arriving at the emergency department (ED) or staying in hospital [1, 2]. Early detection and protocol-based care (PBC) of sepsis with resuscitation, antibiotics, and source control are considered the optimal management strategy [3–6]. Of patients seen in the ED with sepsis, approximately half are transported there by Emergency Medical Services (EMS) [7]. Previous studies have demonstrated that septic patients who arrive by EMS receive treatment rapidly compared to ambulatory patients [8, 9], as with other time sensitive conditions such as myocardial infarction [10, 11].

Paramedic recognition of sepsis is important for the appropriate triage and initiation of early treatment for septic patients transported by EMS. However, identification of sepsis can be challenging for paramedics since they cannot access the laboratory and imaging adjuncts available to physicians in the ED. There is limited information available on how well paramedics are able to identify sepsis compared to emergency physicians (EPs) [12–14], and few screening tools have been developed to assist with prehospital recognition of sepsis [14–17].

Most previous screening tools have focused on detection of patients with severe sepsis, and only one of these tools has been validated to date [15]. Importantly, these screening tools incorporate limited information regarding recent findings of infection in patients and criteria of organ dysfunction remote from the site of infection. To address these limitations, the study team developed a novel prehospital tool to screen for patients with any degree of sepsis. The goal of this study was to evaluate use of this tool by EMS personnel and to compare paramedic detection of septic patients with EP diagnoses.

## 2. Methods

This prospective observational study was of a convenience sample of adult patients (age ≥ 16 years) transported by paramedics to a Canadian tertiary ED. Patients were enrolled into the study by paramedics if they were dispatched as follows: having abdominal pain, having breathing problems, being sick, having unknown problems, being unconscious/fainting, having chest pain, or any case in which paramedics considered sepsis a possible diagnosis. A novel paper-based prehospital sepsis screening tool was developed by the study team. Relevant data points were determined through literature searches related to diagnosis of sepsis, in addition to consensus or guideline statements. The prehospital sepsis screening tool is available in Supplementary Material available online at http://dx.doi.org/10.1155/2016/6717261.

The content of the tool relied heavily upon sepsis criteria definitions developed by the 2001 SCCM/ESICM/ACCP/ATS/SIS sepsis definitions conference [18]. Possible data elements were vetted through use of a Delphi approach with provincial leaders in paramedicine, emergency medicine, and critical care medicine. The tool was evaluated by a focus group of 40 EMS paramedics to obtain feedback and ensure that data elements could be obtained in the prehospital environment without delaying the implementation of the current standard of care to patients. Prior to study initiation, paramedics attended a 3-hour training session and received instruction on sepsis including definitions, epidemiology, basic pathophysiology, identification, and optimal management of septic patients.

In this Canadian provincial ground ambulance system, most paramedics are primary care paramedics (PCPs) or advanced care paramedics (ACPs) (http://novascotia.ca/dhw/ehs/international-applicants/levels-of-paramedics.asp). PCP training is one to two years in length. PCPs can provide basic level emergency care, including performing semiautomated external defibrillation, oxygen administration, intravenous therapy, cardiac monitoring, and administration of select medications. ACP training is 18 to 36 months in length. ACPs are able to provide advanced level emergency care, including a broad array of emergency medications, advanced airway management, and invasive interventions. Training requirements follow the National Occupational Competency Profile (http://www.paramedic.ca/site/nocp?nav=02).

Immediately following transport to hospital and transfer of care to the ED, paramedics were prompted by the EMS dispatch center to complete the prehospital sepsis screening tool. The tool was used to collect data on patient history, vital signs, recent findings (within past 10 days), signs and symptoms, criteria for organ dysfunction remote from the site of infection, and presence of fever. The determination of whether a patient had a fever was based on self-reporting by the patient or the presence of symptoms such as abnormally warm forehead/neck, flushed face, lethargy, and body aches; thermometers were not routinely used by EMS personnel at the time of this study. Based on data collected using the tool, paramedics made a determination of whether or not the patient had any degree of sepsis which was compared to blind, independent documentation of diagnoses by attending EPs.

The EPs used a study ED screening tool to indicate if patients were septic, which served as the gold standard for this study. If the EP did not mark whether or not the patient had sepsis, the final EP diagnosis recorded on the patients' medical record was obtained through retrospective review of the electronic ED database and study investigators determined if the final diagnosis indicated that the patient had sepsis. Data was entered into a dedicated study database (Microsoft® Access, Redmond WA). Analysis was conducted in IBM SPSS Statistics Version 21 (IBM, Armonk NY) [19]. Simple descriptive statistics were used to describe characteristics of paramedics and the patient cohort. Specificity, sensitivity, accuracy, positive and negative predictive value, and likelihood ratios were calculated with 95% confidence intervals. This study was approved by the Institutional Review Board.

## 3. Results

**Figure 1:**
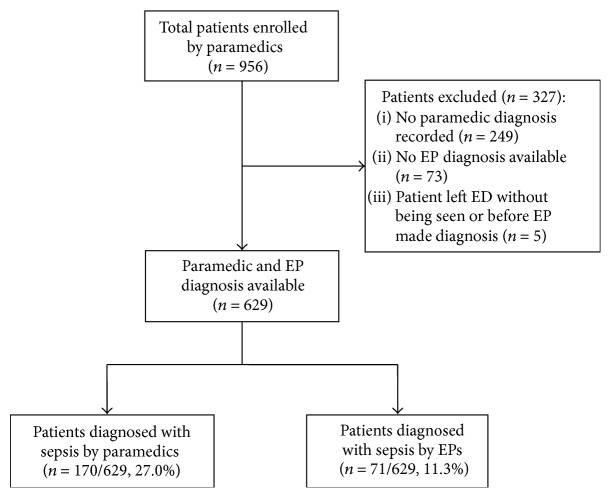
Selection of study participants and identification of sepsis by paramedics and EPs. ED, emergency department; EP, emergency physician.

Overall, 956 patients were enrolled by paramedics during the study period. A flow diagram outlining the selection of study participants and recognition of sepsis by paramedics and EPs is shown in Figure 1. We excluded 327 cases with no paramedic diagnosis recorded (*n* = 249), no EP diagnosis available (*n* = 73), or if the patient left the ED without being seen or before the EP diagnosis was made (*n* = 5). The remaining 629 (65.8%) cases were included in the final analysis.

The certification level of paramedics who participated in the study is shown in Table 1. In most cases, the EMS personnel were an ACP (43.8%) or a PCP (37.4%). Characteristics of the patients seen by paramedics are described in Table 2. Patients commonly presented with fever (27.9%) and with a recent infection within the prior 10 days (25.2%). Paramedics identified 170/629 (27.0%) patients as septic, while EPs identified sepsis in 71/629 (11.3%) patients. Disagreement in the diagnosis of sepsis often occurred in patients ultimately diagnosed with pneumonia, cellulitis, urinary tract infection, peritonitis, and renal failure.

Table 3 shows paramedic identification of sepsis and EP diagnosis of sepsis as a contingency table. The sensitivity and specificity of paramedic diagnoses of sepsis compared to EP diagnoses were 73.2% (95% CI 61.4–83.0) and 78.8% (95% CI 75.2–82.2), respectively. Accuracy was 78.2% (492/629, 52 true positive, 440 true negative). The positive predictive value was 30.6% (95% CI 23.8–38.1), and the negative predictive value was 95.9% (95% CI 93.6–97.5). The positive likelihood ratio was 3.46 (95% CI 2.80–4.29), and negative likelihood ratio was 0.34 (95% CI: 0.23–0.50).

## 4. Discussion

This prospective study assessed the ability of paramedics to recognize septic patients using a novel prehospital sepsis screening tool. Compared with the EPs' diagnosis, we observed that paramedics were able to identify septic patients with 73.2% sensitivity, 78.8% specificity, and 78.2% accuracy using this tool. This study provides evidence that paramedics can identify sepsis with reasonable accuracy in the EMS setting. Increasing paramedic awareness of the importance of early recognition through directed training is critical to reducing the burden of sepsis-related morbidity and mortality.

Few previous studies have examined the ability of prehospital personnel to recognize sepsis in adult patients. Using presence of both an ED report of acute infection and patient admission as their reference standard, one prospective study employed a questionnaire to examine the ability of EMS providers to identify serious infection and reported a sensitivity of 31% (95% CI 17–50) and specificity of 93% (95% CI 87–96) [20]. Other studies have found the sensitivity of sepsis identification by prehospital personnel to be 70% or higher [21, 22], similar to the results of our study.

There are limited reports of validated screening tools for recognition of sepsis in the prehospital setting. One tool recently developed and validated is the prehospital severe sepsis (PRESS) screening tool which involves a score calculated from EMS data on 6 risk factors [15]. Using the PRESS tool in the setting of an urban public hospital, prehospital recognition of severe sepsis by EMS staff had a sensitivity of 86% and a specificity of 47% [15]. The Sepsis Alert Protocol is another screening tool for EMS providers and was found to accurately identify severe sepsis in 47.8% of cases in a pilot study [14]. Unlike these tools, our prehospital screening tool was designed to identify patients with any degree of sepsis, not only those with severe sepsis. Comparing the accuracy of our tool with these other tools is difficult due to variations in study designs, institutional settings, EMS services, and populations served. Additional validation is required before these tools can be recommended for widespread clinical use.

Our study evaluated the ability of paramedics to identify sepsis using a prehospital screening tool and found promising levels of accuracy, sensitivity, and specificity (>70%). These findings are significant in order to capitalize on early alerts to further reduce the time window to treatment initiation [23]. Additional areas for future research may involve integrating the use of biomarkers and evaluating the impact of moving ED treatments earlier into the EMS phase of care. Another opportunity that is yet unexplored is the identification of patients who may have sepsis by EMS dispatchers [24]. There is tremendous potential to reduce the morbidity and mortality associated with sepsis by developing and validating new tools for early recognition and management of the septic patient.

This study has the limitations of an observational study including possible selection and information bias. This study was conducted at a single academic tertiary care center (65,000 ED visits per year) with a single EMS service that takes care of urban, suburban, and rural populations; thus, our findings may not be generalizable to other centers. While we did capture paramedic certification level, data on number of years of experience for paramedics and EPs was not collected. While this study is limited because the reference standard was EP diagnosis instead of a more definitive marker, this reference was chosen as EP diagnosis is the sentinel trigger of PBC at our institution. Another limitation of this study is the number of potentially eligible patients that were excluded, mostly because paramedics did not complete the diagnosis on the study form. At the time of the study, this EMS system did not use thermometers or point of care lactate testing, which may limit generalizability to settings that do use these diagnostic tools.

## 5. Conclusion

Using a novel screening tool, paramedic identification of sepsis in this limited single center study cohort revealed a greater specificity than sensitivity in comparison to EP diagnosis. Further research is required to validate the use of this sepsis screening tool in the prehospital setting.

## Supplementary Material

A prehospital sepsis screening tool was developed by the study team. Paramedics were prompted to complete this paper-based tool immediately following transfer of patient care to the emergency department. Using the tool, paramedics entered data on patient history, vital signs, recent findings within the past 10 days, signs and symptoms, criteria for organ dysfunction remote from site of infection, and presence of fever. Based on this data, paramedics indicated on the tool whether or not the patient had any degree of sepsis.

## Figures and Tables

**Table 1:** Paramedic certification level.

	Frequency
Advanced care paramedic	257 (43.8)
Primary care paramedic	219 (37.4)
Intermediate care paramedic	97 (16.6)
Critical care paramedic	13 (2.2)

Parentheses denote percentage.

*n* = 586 responses.

**Table 2:** Patient characteristics.

	Frequency
Patient history^1^	
Diabetes mellitus	117 (21.1)
Oral steroids or chemotherapy in last 6 weeks	38 (6.8)
Chronic renal failure	23 (4.1)
Organ/tissue transplant	5 (0.9)
Human immunodeficiency virus	1 (0.2)
None of the above	394 (71.0)
Vital signs, mean [SD]	
Respiratory rate	21.6 [8.3]
Heart rate	90.9 [22.6]
Systolic blood pressure^2^	132.3 [29.5]
Diastolic blood pressure^3^	72.8 [14.8]
Blood sugar^4^	8.1 [3.3]
Fever at present^5^	
Yes	163 (27.9)
Recent finding of any infection^6^	
Yes	155 (25.2)
Signs and symptoms of infection^7^	
Pulmonary, cough/hypoxia	170 (27.5)
Abdominal tenderness	81 (13.1)
Soft tissue swelling/redness/pain	62 (10.0)
Altered sensorium	56 (9.0)
Urinary, foul/puss	52 (8.4)
Meningitis, stiff neck	5 (0.8)
None of the above	259 (41.8)
Organ dysfunction criteria^8^	
Diaphoresis	47 (7.8)
Cool peripheral limbs	38 (6.3)
Systolic blood pressure < 90 mmHg	36 (6.0)
Knee mottling	1 (0.2)
Periumbilical mottling	0 (0)
None of the above	498 (82.7)

SD: standard deviation

Brackets denote standard deviation. Parentheses denote percentage.

^1^Paramedics could make more than one selection for patient history, *n* = 555 responses.

^2^ *n* = 627 responses.

^3^Diastolic blood pressure unavailable when palpation was required, *n* = 554 responses.

^4^Blood sugar was often unavailable and only collected in nondiabetic patients, *n* = 446 responses.

^5^Paramedic determination of whether patient had a fever at present, *n* = 584 responses.

^6^Paramedic finding of any infection within prior 10 days, *n* = 614 responses.

^7^Any sign or symptom of infection (both present and new). Paramedics could make more than one selection, *n* = 619 responses.

^8^Any criteria of organ dysfunction present and remote from site of infection that were not chronic conditions. Paramedics could make more than one selection, *n* = 602 responses.

**Table 3:** Contingency table comparing paramedic identification of sepsis with emergency physician diagnosis of sepsis.

	Emergency physician diagnosis of sepsis	
	Yes	No
Paramedic identification of sepsis		
Yes	52	118
No	19	440
