# Supplementary material for: Paramedic Recognition of Sepsis in the Prehospital Setting: A Prospective Observational Study
Source: Emerg Med Int. 2016 Mar 9;2016:6717261. doi: 10.1155/2016/6717261 (PMC4804076; doi:10.1155/2016/6717261)
Supplement: Supplementary file 1 — A prehospital sepsis screening tool was developed by the study team. Paramedics were prompted to complete this paper-based tool immediately following transfer of patient care to the emergency department. Using the tool, paramedics entered data on patient history, vital signs, recent findings within the past 10 days, signs and symptoms, criteria for organ dysfunction remote from site of infection, and presence of fever. Based on this data, paramedics indicated on the tool whether or not the patient had any degree of sepsis. [file 6717261.f1.pdf]

EHS Paramedic Sepsis Screening Tool for Central Region: Trip Destination to QEII HSC ED only

Instructions: Please check off any content that applies. Enter actual numbers where applicable. Please complete and separate the top portion and place into the ED Research box and clip lower portion to the patient chart. Thank you.
Please page your supervisor or John Kasemets (478-7624) if you have any questions

☐**PCP** ☐**ICP** ☐**ACP** ☐**CCP** **MIN**_______________ **PCR #**_______________ **HUN#**_______________

1. **HISTORY:** ☐DM ☐HIV ☐CRF ☐Oral steroids or chemo in last 6 weeks ☐Organ/tissue transplant ☐None of the above

2. **VITAL SIGNS:** RR_____ HR _____ B/P______ Does this patient have a fever at present? (Circle) Yes No

Blood sugar (for non-diabetic) __________

3. **RECENT FINDINGS (Within last 10 days):**

☐ Implantable device infection
☐ Bloodstream catheter infection
☐ Acute abdominal infection
☐ Recent hospital stay
☐ Endocarditis
☐ Urinary tract infection
☐ Recent ED visit
☐ Skin/soft tissue Infection
☐ Pneumonia
☐ Meningitis
☐ Wound infection
☐ None of the above
☐ Bone/joint infection
☐ Recent antibiotics

4. **SIGNS & SYMPTOMS: Are any of following signs & symptoms of infection both present and new?**

☐ Meningitis- stiff neck
☐ Abdo tenderness
☐ Altered sensorium
☐ Soft tissue swelling/redness/pain
☐ Pulmonary- cough/hypoxia
☐ Urinary- foul/puss
☐ None of the above

5. **Are any of the following organ dysfunction criteria present and remote from the site of the infection that are not chronic conditions?**

☐ Periumbilical mottling
☐ SBP < 90mm/hg
☐ Diaphoresis
☐ Knee mottling
☐ Cool peripheral limbs
☐ None of the above

6. **Consider other forms of shock** (check if **NOT** present)

☐ Cardiogenic shock (no ischemic chest pain, CHF, or 12 lead changes - if available)
☐ Hypovolemic shock
☐ Evidence of major trauma
☐ Evidence of major bleeding, ex GI
☐ Anaphylaxis (no evidence of allergic trigger, swelling, hives)
☐ Evidence of toxic exposure (drug overdose or chemical exposure)

Does this patient have sepsis? (Circle) Yes No Time to complete this form:____________(minutes)

EHS Emergency Health Services

DALHOUSIE UNIVERSITY Inspiring Minds

Capital Health
